# The Impact of Artificial Intelligence (AI) on Midwifery Education: A Scoping Review

**DOI:** 10.3390/healthcare12111082

**Published:** 2024-05-24

**Authors:** Angela Kranz, Harald Abele

**Affiliations:** 1 Section of Midwifery Science, Institute of Health Sciences, University of Tübingen, 72076 Tübingen, Germany; harald.abele@med.uni-tuebingen.de; 2 Department for Women’s Health, University Hospital Tübingen, 72076 Tübingen, Germany

**Keywords:** midwifery, higher education, artificial intelligence

## Abstract

As in other healthcare professions, artificial intelligence will influence midwifery education. To prepare midwifes for a future where AI plays a significant role in healthcare, educational requirements need to be adapted. This scoping review aims to outline the current state of research regarding the impact of AI on midwifery education. The review follows the framework of Arksey and O’Malley and the PRISMA-ScR. Two databases (Academic Search Premier and PubMed) were searched for different search strings, following defined inclusion criteria, and six articles were included. The results indicate that midwifery practice and education is faced with several challenges as well as opportunities when integrating AI. All articles see the urgent need to implement AI technologies into midwifery education for midwives to actively participate in AI initiatives and research. Midwifery educators need to be trained and supported to use and teach AI technologies in midwifery. In conclusion, the integration of AI in midwifery education is still at an early stage. There is a need for multidisciplinary research. The analysed literature indicates that midwifery curricula should integrate AI at different levels for graduates to be prepared for their future in healthcare.

## 1. Introduction

Artificial Intelligence (AI) is transforming various industries and the healthcare industry is expected to benefit significantly [1]. By sorting and analysing huge amounts of research, clinical and patient data, AI technologies in healthcare are capable of recognizing patterns to enhance knowledge generation and decision-making [2,3,4].

There are numerous definitions of AI [5] and it encompasses different computational approaches [6]. Essentially, AI is a technology that enables a computer system to learn, think, perceive, reason, communicate and ultimately make decisions in a way that is similar to or perhaps even better than humans [3,7]. Software and hardware systems work towards a goal in both physical and digital dimensions, collecting, structuring, analysing and interpreting data. Knowledge is derived from these data, and the best course of action to achieve the given goal is determined [8]. The objective is to imitate and extend human intelligence [9,10].

Predictions suggest that AI healthcare technologies will revolutionize healthcare in the coming decades [3]. This will lead to new roles and models of care emerging for healthcare professionals [11]. Consequently, educational requirements and competences of healthcare professionals will need to be adapted [3].

Midwives worldwide number approximately 1.9 million, making them an essential professional group in the healthcare sector [12]. There are various potential applications of AI for this profession, along with associated risks and opportunities [10]. AI allows for better informed decision-making [2,3,4] and it is expected to contribute to better patient outcomes as well as a more efficient healthcare system [4]. The ethical and moral dimensions are particularly important in the age of AI [4]. Initial studies already show that the midwifery profession is sceptical of AI technologies, especially in terms of clinical decision-making, cultural values, and ethics [13]. O’Connor recommends including AI in midwifery curricula [6], allowing midwives to prepare for their professional careers and take the lead in AI initiatives in healthcare [6]. Such initiatives refer to new developments in the field of AI in healthcare as well as in clinical research. By taking the lead in these initiatives, midwives will be able to contribute to evidence-based practice and policy [6].

Midwifery educators are expected to teach such technical topics [6], while studies in the related field of nursing have shown that educators face difficulties in teaching them [14]. To address this issue, it is advisable to involve professionals from the fields of computer science or other related AI disciplines [6].

AI research in healthcare education has grown rapidly over the last decade [9], particularly in the context of higher education [15]. Previous research has mainly focused on the disciplines of medicine [9,16,17], nursing [3,18,19] and on AI in higher education in general [15,20,21]. AI has, and will have, a major impact on higher education across all disciplines, ranging from social impacts, such as those on teaching and learning, to legal issues [20].

Some of the major risks related to AI in midwifery education are misinformation and plagiarism [22]. Whereas AI-generated content can be detected by several reliable AI tools [23], privacy concerns are still a major threat when using AI technologies such as ChatGPT (Generative Pretrained Transformer [24]) in education. These technologies use the data entered to train the model itself [25]. As AI technologies are based on the quality of their training data, it is not clear whether high-quality data are available in the field of midwifery [4].

A systematic review of AI in medical education found that students had positive attitudes towards AI-related courses. The review also highlighted the fact that the research on AI in medical education has been of poor quality so far [9]. According to another review of AI in nursing education, there is an urgent need to adapt curricula to the age of AI, and educators should receive special training to meet these needs [3].

However, the current evidence on the use of AI in midwifery is limited, particularly in the field of education [6,10]. A review of AI in nursing and midwifery was recently published, highlighting its potential contributions to education, research, clinical practice and policy. The review recommends integrating AI into midwifery education [10]. Initial scientific discussions already consider the use of technologies such as ChatGPT in higher midwifery education [4]. Still, further research is required on AI in midwifery, especially in midwifery education. This is necessary to investigate potential opportunities and challenges, as well as potential outcomes [6,10].

To outline the current state of research and identify existing gaps in knowledge of AI and midwifery education, we conducted a scoping review. The following research question was formulated: How does AI impact midwifery education? The objectives of this scoping review were to analyse the potentials, risks, integration and the role of educators of AI in midwifery education, as well as the applications of AI in higher education.

## 2. Methods

The purpose of scoping reviews is to explore the key characteristics of a research area [26]. They provide an overview of the current state of evidence [27]. This is particularly appropriate for this research question, as it is a relatively new area of research and the existing evidence needs to be structured [27]. The scoping review followed the framework of Arksey and O’Malley [28], the update by Levac et al. [29], the PRISMA-ScR [26] and other recommendations [27]. Therefore, the following steps were followed: (1) identifying the research question; (2) identifying relevant studies; (3) study selection; (4) charting the data; and (5) collating, summarizing and reporting the results [28,29].

(1) Identifying the research question

The research question was formulated using the PCC scheme, which stands for population, concept, and context [26,30]. The necessary components are presented in Table 1. In consequence, the following research question arose: how does AI impact midwifery education?

(2) Identifying relevant studies

A comprehensive search strategy was used to identify the relevant literature. Specific keywords were searched in different databases to retrieve relevant articles. Various search terms were devised with the use of wildcards. Using Boolean operators, the search terms were combined into search strings. The literature search was conducted using the databases PubMed and Academic Search Premier and Table 2 shows the search strategy as described.

(3) Study selection

To select relevant studies, the first step was to remove duplicates. Therefore, the search results of the search strings (Table 2) were imported into one Microsoft Excel file. Duplicates are identified using the command “double values”. After removing duplicates, the articles were imported into the software Mendeley Reference Manager v2.115.0, screening their title and abstract. After excluding articles that did not match the research question, full-text articles were screened for assessing eligibility. Again, Mendeley was used to manage the full-text article screening, checking the articles for assessing inclusion criteria, leading to a final inclusion of articles in the review.

The literature search and selection were conducted in May 2024. The study selection was conducted by two independent researchers. If the authors disagreed on the inclusion or exclusion of an article, this was discussed in a personal meeting until consensus was reached.

Table 3 shows the inclusion criteria. As the topic of this scoping review was contemporary, only literature from 2021 onwards was included. The study type was not restricted, to enable the most sensitive search possible. Only publications published in English were included.

(4) Charting the data

The data extraction was based on the objectives and research question of the scoping review, providing a descriptive summary of the results. The following characteristics were extracted from the included literature and summarized in a Table: author, year, title, country, objective, methods, summary of the results, and recommendations for midwifery education.

(5) Collating, summarizing, and reporting the results

In this phase, information was extracted from six articles. The authors of these articles were from eight different countries, whereas two of the articles were reviews including literature from several countries. Based on the defined characteristics in step 4, this enabled the defined research question in the step 1 question to be answered. The articles are summarized, focusing on midwifery education.

## 3. Results

**Figure 1:**
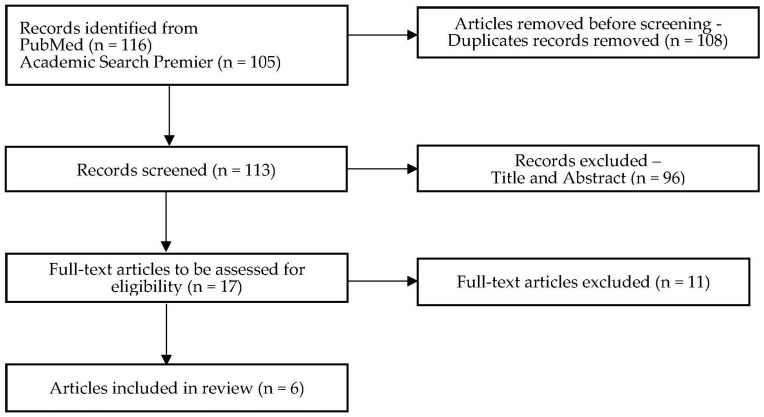
PRISMA flow diagram.

The literature search identified 221 records across two databases, of which 108 duplicates were removed. After screening *n* = 113 records for their title and abstract, *n* = 17 articles remained for assessing eligibility. After the exclusion of *n* = 11 articles as they did not meet the inclusion criteria, *n* = 6 articles were included in the review. The search process is illustrated in the PRISMA flow diagram (Figure 1).

### 3.1. Literature Synthesis

Details of the literature synthesis are shown in Table 4. The results indicated that AI presents both opportunities and challenges for midwifery practice and education which can be categorized into the following areas:

#### 3.1.1. Potentials of AI in Midwifery

The potentials of AI in midwifery are emphasized by the analysed articles. These include improving the quality of care [13], safety [31], optimizing the decision-making process [10,13], and simplifying administrative tasks [13], as well as increasing precision and productivity [32]. As a result, midwives have more time for patient care [13]. Moreover, improved patient outcomes and their prediction are also mentioned [4,10], as well as a more efficient healthcare system in general [4]. However, there is a discrepancy between the researched benefits and the actual potentials of AI in midwifery [10].

#### 3.1.2. Risks of AI in Midwifery

To provide a comprehensive overview of AI in midwifery, the articles also highlight the possible risks of AI in midwifery. The use of AI-generated content in clinical practice is questioned, due to the quality of the training data on which AI technologies are based on [4,10]. Moreover, ethical, legal and data protection concerns are emerging [10,13], as well as concerns regarding the loss of human interaction [13] and other social implications of AI [10].

#### 3.1.3. Applications of AI in Higher Education

Technologies such as ChatGPT can significantly improve teaching by enabling personalized learning materials and assessments, creating simulated clinical scenarios and providing immediate individual feedback [4]. AI can support students in research and writing skills [4], as well as providing tutoring [31]. The use of resources can be improved by using AI in education [31]. However, there are also challenges, such as the threat of AI to academic integrity (e.g., plagiarism and questioning traditional assessment methods) [4]. Potential risks include the generation of false information, threats to privacy, security, and confidentiality [31]. The impact of AI on online and off-campus learning is expected to be significant, leading to changes in traditional teaching strategies [31].

#### 3.1.4. Integration of AI in Midwifery Curricula

Having considered the potential benefits and risks, all the articles conclude that AI needs to be integrated into midwifery curricula [4,6,10,13,31,32]. Midwives should be able to participate in and lead AI initiatives, as well as conduct AI-related research in healthcare [6,10,32]. In order to successfully incorporate AI into midwifery practice, midwives need to receive appropriate education [13] and it is important that midwifery students are prepared for their future careers, as AI plays a significant role in healthcare [4,31]. Furthermore, integrating AI into midwifery curricula can increase midwives’ acceptance of this technology [13]. Both undergraduate and postgraduate programs should include AI training [10] and graduates as well as midwives in practice should be able to handle AI [10]. Experts from AI-related disciplines as well as technology companies should be involved to ensure a comprehensive approach toward AI education in midwifery [6,10]. In general, students need to be educated about the ethical, legal and technical dimensions of AI technologies in midwifery [4,6,10,13].

#### 3.1.5. The Role of Midwifery Educators

The use of AI in midwifery education allows educators to focus on students’ needs and more meaningful interactions [4]. Educators should exemplify the use of AI, both on campus and in professional practice [31]. It is highlighted that midwifery educators play a key role in integrating AI in midwifery, which is why they need to be trained in AI-related topics [6,10]. Midwifery educators can receive support from AI experts [6,10], as well as high-quality scientific AI literature [6].

## 4. Discussion

### 4.1. Summary of the Results

The literature synthesis of six articles indicates a profound impact of AI on midwifery education [4,6,10,13,31,32], thus providing an answer to the research question regarding the impact of AI on midwifery education. These impacts can be categorized into the following areas: potentials and risks of AI in midwifery, applications of AI in higher education, integration of AI in midwifery curricula, and the role of midwifery educators. Given the potential benefits and risks of implementing AI in midwifery practice, it is crucial for midwifery graduates and practitioners to receive appropriate training. All the articles conclude that AI-related topics should be integrated into midwifery curricula [4,6,10,13,31,32] to enable midwives to actively participate in and lead AI initiatives in healthcare [6,10,32]. In addition to educating midwives on AI applications in midwifery practice, the use of AI technologies such as ChatGPT in higher education is highly relevant [4]. The impact of AI is changing traditional teaching strategies and leading to personalized learning materials and assessments [4,31]. However, there are some threats, such as academic integrity, which need to be addressed [4]. Midwifery educators play a key role in all these possible applications. They need to be trained in AI-related topics and supported by AI experts [6,10].

### 4.2. Potentials of AI in Midwifery

The potentials of AI extend far beyond midwifery, as it affects the healthcare sector in general [1] and leads to a more efficient healthcare system [4]. The literature analysed in this scoping review highlights the potentials of AI in midwifery by improving the quality of care, safety, and decision-making, as well as providing more time for care [4,10,13,31]. Further literature suggests that AI can improve the health and well-being of pregnant women by automatically analysing their emotions. Although there are few studies in this area, a review shows that this is a promising field of AI in midwifery, obstetrics and gynaecology [33]. Furthermore, AI technologies can improve universal healthcare and reduce healthcare variability by accurately monitoring health status during pregnancy, recognizing risk factors and contributing to automated data analysis for more efficient decision-making [33]. Further studies indicate that AI-related technologies enable midwifery-specific advancements such as improved diagnosis, pregnancy risk assessment and foetal monitoring. The primary goal should be to assist human experts in their work [34]. This is supported by the literature analysed here, as midwives expressed a desire for AI to support them in their daily work [13]. Overall, further research is needed in several areas for AI to be successfully implemented in midwifery [33,34]. The analysed literature supports the integration of midwives into AI-related research [10].

AI technologies in healthcare are regulated by laws and standards to handle the secure and ethical use of patient data. For example, the Health Insurance Portability and Accountability Act (HIPAA) in the United States is a central component of data privacy in healthcare, setting strict rules for managing electronic health records and other medical data [35]. AI itself is a powerful tool to defend data privacy in healthcare and enhance the security of sensitive patient data. By analysing user behaviour and data access patterns, AI can identify inadvertent actions in real-time and potential threats can be identified at an early stage, allowing the implementation of measures to prevent security breaches. AI solutions facilitate the encryption and anonymization of large numbers of data in the healthcare sector, enabling researcher and healthcare professionals to safely use big datasets [35].

### 4.3. Risks of AI in Midwifery

However, the transition from theory to practice poses several potential risks that could undermine the trust of healthcare professionals and patients in AI-related systems [34]. The examined studies reveal a lack of trust in these systems and scepticism among midwives [13], as well as the discussion of the ethical and legal implications of AI for midwifery [10,34]. The use of large and high-quality training datasets is crucial for accurate results in practice [34]. This is also highlighted in the analysed articles, which recommend that midwives themselves should collect high-quality patient and health data to enhance care through AI technologies [10]. To effectively manage the potentials and risks of AI in midwifery, students and practitioners require adequate education [10].

### 4.4. Applications of AI in Higher Education

AI has the potential to transform the practice and experiences of students in higher education. AI can be applied at different levels in higher education, and at an institutional level AI can help with student selection and predict dropout and group behaviour trends, enabling new directions and realignments of study programs. Another use case for AI in higher education is directly linked to teaching and learning [3]. The focus lies on using AI for personalization and adapting learning to the needs of groups and individuals, and it aims to support the individual learning process [21]. In their review of the potential applications of AI in higher education, Crompton and Burke summarized the following areas: assessment and evaluation (e.g., academic progress assessment), predication (e.g., identifying students who need support), AI assistant (e.g., student support), intelligent tutoring systems (e.g., learning strategies tailored to students’ needs) and managing student learning [15].

Some important use cases of AI in higher education are language models such as ChatGPT, which increasingly create realistic scientific texts. Studies in the field of medicine demonstrate that ChatGPT can produce high-quality scientific abstracts, which are difficult for reviewers to distinguish [36]. To address this issue, several reliable AI tools have been developed to recognize the texts generated by ChatGPT, and the utilization of such AI tools can diminish the probability of students and academics submitting texts solely generated by ChatGPT [23]. However, it is unclear how ChatGPT deals with misinformation and plagiarism [22], and privacy concerns and bias are also discussed as potential risks when using ChatGPT in a scientific context [37]. One of the key concerns when using ChatGPT in an academic context is the potential for the data entered to be used to train the model itself; therefore, it is important to use AI technologies such as ChatGPT responsibly and with caution and to take appropriate measures to protect potential risks to user privacy [25].

To prevent academic fraud and prepare students for the use of ChatGPT and other AI technologies, it is recommended that faculty members proactively and ethically use and provide education about these tools [37]. These trends can be emphasized by the studies examined in the present review. Models such as ChatGPT can be beneficial in midwifery education, from individualized learning experiences to the promotion of academic skills. However, plagiarism as the greatest risk when using ChatGPT is also discussed in midwifery-related articles. Since AI models depend on their training data, the question arises as to whether sufficient and high-quality data are available in the field of midwifery [4]. Studies indicate that insufficient data quality in midwifery can potentially increase health inequalities [6]. Overall, it is evident that the regulation of AI in higher (midwifery) education is necessary [4,22].

### 4.5. Integration of AI in Midwifery Curricula

The developments in the field of AI are not only changing higher education, but are also impacting on professional working environments and is important that students are prepared for a future where AI plays a significant role [20]. AI technologies are emerging especially in the field of medicine and related areas, such as nursing and midwifery. Initial studies indicate that students are interested in learning about AI in medicine, but few faculties worldwide educate about the application of AI in healthcare [17]. Other studies in the field of nursing indicate an urgent need to adapt curricula to the age of AI, for graduates to use AI safely and efficiently [3].

### 4.6. The Role of Midwifery Educators

Educators are uncertain about how to pedagogically use AI as well as how to apply it meaningfully to teaching and learning in higher education, and there is a lack of educational theories connecting with the effective application of AI. The ethical and social dimensions of integrating AI into teaching and learning is also a concern for educators [38] and studies have shown that nursing educators have concerns about the impact of AI on their workload and their role in the teaching process. Therefore, the appropriate training of educators is crucial, and AI should enhance the learning process for educators to have time for more complex teaching tasks [39]. The literature examined in the present paper in the field of midwifery supports the described developments of AI technologies in higher education and nursing education and studies emphasize that midwifery educators have a responsibility to engage with the risks and opportunities of AI in both education and healthcare practice. Midwifery educators need to be capable of supporting students in safely using AI in midwifery [31]. Faculties in midwifery are encouraged to seek support from computer technologies and AI-related disciplines, to adapt their curricula and train midwifery educators [6,10].

The application of AI in higher education is still in an early stage. However, there is a need for midwifery curricula to integrate AI, for their graduates to be prepared for the age of AI [6] as well as for further multidisciplinary research [21]. This includes computer scientists, data experts, experts in informatics and social informatics, sociologists, psychologists, lawyers, anthropologists, educators [21] and, of course, midwives and medical experts [6]. Research should concentrate on questions related to the practical applications of AI in higher education. For example, one area of interest is the impact of AI on learning outcomes [21].

### 4.7. Limitations

This scoping review has several limitations as well as strengths. The literature search was open and sensitive, but it is possible that relevant literature was overlooked. Another limitation is that the quality of the included literature was not evaluated. Furthermore, the analysed research area is rapidly developing and relatively new, and at the time of the literature search there were limited studies on the given topic. As a result, this paper can serve as a general overview and a descriptive presentation of the current state of research, but cannot provide any recommendations for practice and policy due to the lack of literature and primary data. Despite these limitations, it is important to note that the scoping review is based on a comprehensive literature search, as well as on several recommendations that were followed [26,27,28,29].

### 4.8. Future Implications for Research

Based on the scoping review, there are existing gaps in research that need to be addressed, whereas it needs to be stated that the method of a scoping review does not allow recommendations to be made for practice and policy [26].

A systematic review about AI in midwifery education is recommended to analyse more studies about AI in midwifery education, as well as to analyse the quality of the included studies. Future research could focus on more specific areas of AI in midwifery education, such as how midwifery faculties could implement AI in their curricula or what effects AI technologies have on personalized, learner-centred education. Comprehensive and high-quality systematic reviews about AI in midwifery education are needed to identify clear future research possibilities for practice and policy.

## 5. Conclusions

The scoping review identifies six articles, all emphasizing the significant impact of AI on midwifery practice and education. The recommendations of the analysed articles for integrating AI into midwifery curricula are based on multiple applications and the associated risks and opportunities of AI in healthcare practice [4,6,10,13,31,32]. Additionally, AI technologies can change teaching practices in higher education and enable personalized, learner-centred education [4,31]. However, there are still open questions such as academic integrity when using language models such as ChatGPT in higher education [4]. Midwifery educators are seen as central, as they serve as role models for students and provide education on such technical topics [6,10]. The related fields of nursing and medicine are making further progress in researching AI in their education [3,9,11,16,17,18,19,22,36,39]. This can serve as an orientation for midwifery; nevertheless, there is a need to investigate the integration of AI into midwifery education at various levels, involving multidisciplinary research teams from computer, medical and social sciences and other relevant fields of knowledge [6,21].

## Figures and Tables

**Table 1:** PCC framework.

P	Population	Midwives
C	Concept	Artificial Intelligence
C	Context	Education

**Table 2:** Search strategy.

Search	Search Date	Search String	Database	Additional Filter: Publication Date	Results
S1	14 May 2024	(midwif*) AND (artificial intelligence*) OR (ChatGPT*) AND (educat*) AND (nurs*)	Academic Search Premier	1 January 2021–1 May 2024	89
			PubMed	1 January 2021–1 May 2024	95
S2	14 May 2024	(midwif*) AND (student*) AND (artificial intelligence*) OR (ChatGPT*) AND (teaching*) AND (nurs*)	Academic Search Premier	1 January 2021–1 May 2024	16
			PubMed	1 January 2021–1 May 2024	21

**Table 3:** Inclusion Criteria.

Articles addressing the elements of PCC (Midwives, Artificial Intelligence, Education)
English articles
Articles published after 2020

**Table 4:** Synthesis of the results.

Author(s)	Year	Title	Country	Objective	Methods	Summary of Results	Recommendations for Midwifery Education
Çitil & Canbay [13]	2022	Artificial intelligence and the future of midwifery: What do midwives think about artificial intelligence? A qualitative study	Turkey	The aim is to explore midwives’ views on the future of AI and midwifery.	Qualitative study with semi-structured interviews	The findings indicate that midwives are optimistic about the potentials of AI. The opportunities for AI in midwifery include the enhancement of care qualityand optimizing decision-making as well as simplifying administrative tasks. This leads to midwives having more time for patient care. However, the study also highlights concerns about AI, particularly about unresolved ethical and data protection issues as well as the potential loss of human interaction in care. Additionally, midwives mention trust issues about AI. In terms of education, the study highlights the fact that appropriate AI education of midwives is necessary to effectively integrate AI into midwifery practice.	The results suggest that AI should be integrated into midwifery curricula to utilize its potential as well as to enhance the acceptance of AI in midwifery.
Irwin et al. [4]	2023	What is ChatGPT and what do we do with it? Implications of the age of AI for nursing and midwifery practice and education: An editorial	Australia	The aim is to inform about the opportunities and challenges of using ChatGPT in nursing and midwifery practice and education.	Editorial	The article demonstrates how ChatGPT and other AI technologies can enhance midwifery education. These technologies enable personalized learning materials and assessments, simulating clinical scenario creation and instant individualized feedback provision to students. Integrating AI into the curriculum allows educators to concentrate more on learners’ needs and engage in meaningful interactions with students. Moreover, students can be assisted in developing research and writing skills. However, integrating AI technologies into midwifery education poses some challenges. One of the main concerns is academic integrity, as ChatGPT can generate high-quality texts. This raises issues regarding plagiarism and traditional assessment methods in higher education. Additionally, ethical concerns arise when using AI-generated content in clinical practice, which depends on the quality of the data used to train them.	The article concludes that midwifery curricula should be adapted to prepare students for a future where AI plays a significant role in healthcare. It is necessary to carefully consider the advantages as well as disadvantages of AI in midwifery practice and education. Through technologies such as ChatGPT, higher education has the chance to enable learner-centred content more than ever before, and to allow for better patient outcomes as well as a more efficient healthcare system in general.
Mather et al. [31]	2023	Nursing and midwifery education: Part 1	Australia	The aim is to inform and encourage midwifery educators about the integration of AI into midwifery education.	Focus article	Midwifery graduates will use AI tools in their future careers, which is why it is important to include AI in midwifery curricula. Educators should model the use of AI on campus and in professional environments, as it is becoming an essential part of the digital identity of future midwives. AI can be used in various ways, ranging from tutoring students to improving safety and quality in healthcare. In education, AI has a major impact on traditional teaching strategies, particularly for online and off-campus learning experiences. However, it is important to consider the potential risks associated with AI, such as generating false information and threats to privacy, security and confidentiality.	AI tools can assist in ensuring efficient resource use and supporting learning, but they cannot replace midwifery care. Educators must continuously develop their students’ AI competences and digital literacy. Midwifery graduates should be capable of ensuring safety and quality in healthcare, including the use of AI technologies.
O’Connor et al. [10]	2021	Artificial intelligence in nursing and midwifery: A systematic review	United Kingdom, Hong Kong, Switzerland, Ireland	The aim is to identify and synthesise the scientific literature on AI in nursing and midwifery. The review focusses on how AI is used in nursing and midwifery education, research, and policy.	Systematic review	The article shows that AI is mainly utilized in clinical practice to provide patient care. The involvement of midwives in AI varies greatly, from actively testing AI to complete exclusion from studies. The potential of AI lies in its ability to predict health outcomes with higher accuracy and support clinical decision-making. However, there is a gap between the actual benefits and the potential of AI. The main risks associated with AI include poor-quality data sets and retrospective data, which limit AI predictions. Additionally, there are ethical, legal, and social concerns surrounding AI. The article emphasizes the importance of integrating AI into midwifery curricula to ensure that midwives are prepared to lead and intervene in digital health initiatives.	Incorporating AI into midwifery education will provide midwives with the necessary skills and knowledge to handle AI in practice. The curricula of midwives should incorporate various aspects of AI. This is relevant for both existing undergraduate and postgraduate midwifery programs. Furthermore, midwifery faculties should invest in their professional development to ensure that midwifery educators are capable of educating about AI-related subjects. The inclusion of other AI-related disciplines and technology companies in midwifery curricula is seen as beneficial.
O’Connor [6]	2022	Teaching artificial intelligence to nursing and midwifery students	United Kingdom	The aim is to encourage midwifery and nursing curricula to include AI.	Editorial	To prepare midwifery students for a future in which AI plays a significant role in healthcare, their curricula should be adapted and evaluated in terms of AI. This will enable students to acquire the necessary skills to actively participate in and lead AI initiatives in healthcare, facilitating their involvement in evidence-based practice and policy. Integrating computer scientists or other AI experts is beneficial for midwifery faculties, as they are faced with challenges teaching technical topics. It is recommended that faculties incorporate high-quality scientific AI content into their curricula. Additionally, students should be provided with insights into the development of AI systems in healthcare, e.g., by technology companies. Incorporating ethical, social and legal implications into AI education is crucial, as the importance of AI extends beyond technical aspects.	To adapt to the rapid evolution of AI, it is urgent to invest in midwifery education and provide clinical midwives with further education opportunities to effectively handle AI in healthcare.
Yakout & Jahlan [32]	2023	Artificial Intelligence: Innovation and Midwifery Education, Practice, And Research in Arab Region; Systematic Review Based Findings	Arab region (Saudi Arabia and Egypt)	The aim is to review the literature on AI innovations in midwifery education, practice and research in the Arab region.	Systematic review	By enabling advanced, precise and efficient healthcare interventions, the article demonstrates the significant potentials of AI in midwifery education, practice, and research. AI innovations provide new learning opportunities in midwifery education and enable advanced, precise, and competency-based learning and teaching. The article emphasizes the need to reform midwifery curricula and integrate AI into midwifery education. The integration of AI into midwifery enables midwives to achieve a higher level of precision and productivity in their clinical practice. This can be promoted through education.	The use of AI in midwifery has the potential to improve education, healthcare, and research. However, it is important to handle ethical considerations carefully, and to continuously evaluate its use to minimize potential risks.

## Data Availability

Not applicable.
